# Evaluation of the OpSens OptoWire III and Novel TAVR Algorithm to Measure Pressure Gradient During TAVR

**DOI:** 10.1016/j.jscai.2022.100309

**Published:** 2022-05-17

**Authors:** Philippe Généreux, Robert M. Kipperman, Jenny S. Placido Disla, Lillian Aldaia, Konstantinos P. Koulogiannis, Leo Marcoff, Anuj Mediratta, James P. Slater, Bledi Zaku, Björn Redfors, Omar M. Abdelfattah, Linda D. Gillam

**Affiliations:** a Gagnon Cardiovascular Institute, Morristown Medical Center, Morristown, New Jersey; b Cardiovascular Research Foundation, New York, New York

**Keywords:** Hemodynamics, transcatheter aortic valve replacement, catheterization, echocardiogram

## Abstract

**Background:**

We aim to establish the degree of agreement related to gradient measurement during transcatheter aortic valve replacement (TAVR) between the OpSens OptoWire III and its new proprietary TAVR algorithm and hemodynamic value derived by catheterization and echocardiogram (transthoracic echocardiogram and transesophageal echocardiogram).

**Methods:**

The current study was a prospective, single-arm, single-center study. All subjects underwent hemodynamic assessment before and after TAVR using standard hemodynamic assessment using 2 pigtails, transthoracic echocardiogram, transesophageal echocardiogram, and the OpSens OptoWire III. The primary end point was the final post-TAVR mean gradient correlation between OpSens OptoWire III and hemodynamic values derived by catheterization.

**Results:**

Between July 2021 and September 2021, 20 patients were enrolled. The median age was 79 [6.5] years, and 9 (45%) patients were female. The mean gradient before TAVR derived by 2-pigtail technique and the mean gradient using the OpSens OptoWire III were similar (35 ± 14 mm Hg vs 35 ± 14 mm Hg, *P* = 1.00), with an absolute mean difference of 2.2 ± 3.5 mm Hg and a strong correlation (*r* = 0.96, *P* < .0001). After TAVR, the mean gradient derived by 2-pigtail technique and the mean gradient using the OpSens OptoWire III were similar (2.2 ± 3.5 vs 2.8 ± 2.7, *P* = .16), with an absolute mean difference of 1.2 ± 1.3 mm Hg and a strong correlation (*r* = 0.89, *P* < .0001).

**Conclusions:**

Hemodynamic assessment derived by the OpSens OptoWire III wire and its new TAVR algorithm demonstrated excellent correlation with measurements derived by 2 pigtails both before and after TAVR. Integration of this new technology within a dedicated TAVR wire with live hemodynamic assessment could bring meaningful value to TAVR operators.

## Highlights

• Gradient measurement during TAVR using the OpSens wire was feasible and safe.
• OpSens wire shows strong correlation with 2-pigtail gradient measurement during TAVR.
• Invasive and echocardiographic gradient measurements were similar before TAVR.
• Discrepancies between invasive and echocardiographic gradients were seen after TAVR.

## Central Illustration

**Figure:**
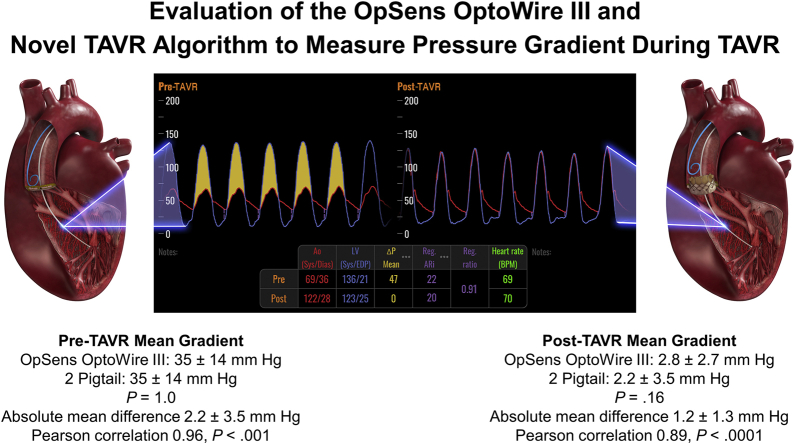

Transcatheter aortic valve replacement (TAVR) has become a well-established procedure, performed mainly without general anesthesia and under conscious sedation.^1^, ^2^, ^3^, ^4^, ^5^, ^6^ The evaluation of the hemodynamic severity of aortic stenosis (AS) before the procedure and evaluation of post-TAVR results have relied mainly on echocardiographic measurements or hemodynamic assessment derived by catheterization.^7^^,^^8^ While the use of invasive hemodynamic assessment through cardiac catheterization (double pigtails and transducers) represents the gold standard in terms of accuracy of measurement both before and after TAVR, its use is cumbersome, time-consuming, and has decreased with the use of transthoracic echocardiogram (TTE)^8^; however, echocardiographic measurements are highly dependent on acquisition technique and patient anatomy and vary based on the type of aortic valve prosthesis used.

Recently, OpSens Inc has developed the SavvyWire, a dedicated 0.035″ left ventricular TAVR wire capable of delivering rapid pacing and providing live pressure gradients paired with a novel dedicated console and TAVR algorithm. The core of this novel technology is built around a fiber optic sensor (Fidela) imbedded in a wire. The aim of the current study is to establish the accuracy and degree of agreement related to gradient measurement during TAVR between the OpSens OptoWire III, a currently available 0.014″ wire embedding the same optical sensor as the SavvyWire, and the new TAVR algorithm and hemodynamic values derived by standard catheterization techniques and standard TTE and transesophageal echocardiographic (TEE) measurements. This study will provide additional data validating the robustness of the OpSens TAVR algorithm using a 0.014″ wire and whether the integration of this new technology within a dedicated 0.035″ TAVR wire with live hemodynamic assessment could bring meaningful value to TAVR operators.

## Methods

### Study population

Patients presenting with severe AS undergoing TAVR using the Edwards SAPIEN 3/SAPIEN 3 Ultra system were considered for enrollment. Key exclusion criteria included (1) hemodynamic instability making the use of additional hemodynamic measurement inappropriate or 24-hour survival unlikely and (2) inability to obtain TTE, TEE, or hemodynamic measurements using standard catheterization techniques. Consented subjects underwent screening procedures and were scheduled for their TAVR procedure.

### Study device

**Figure 1:**
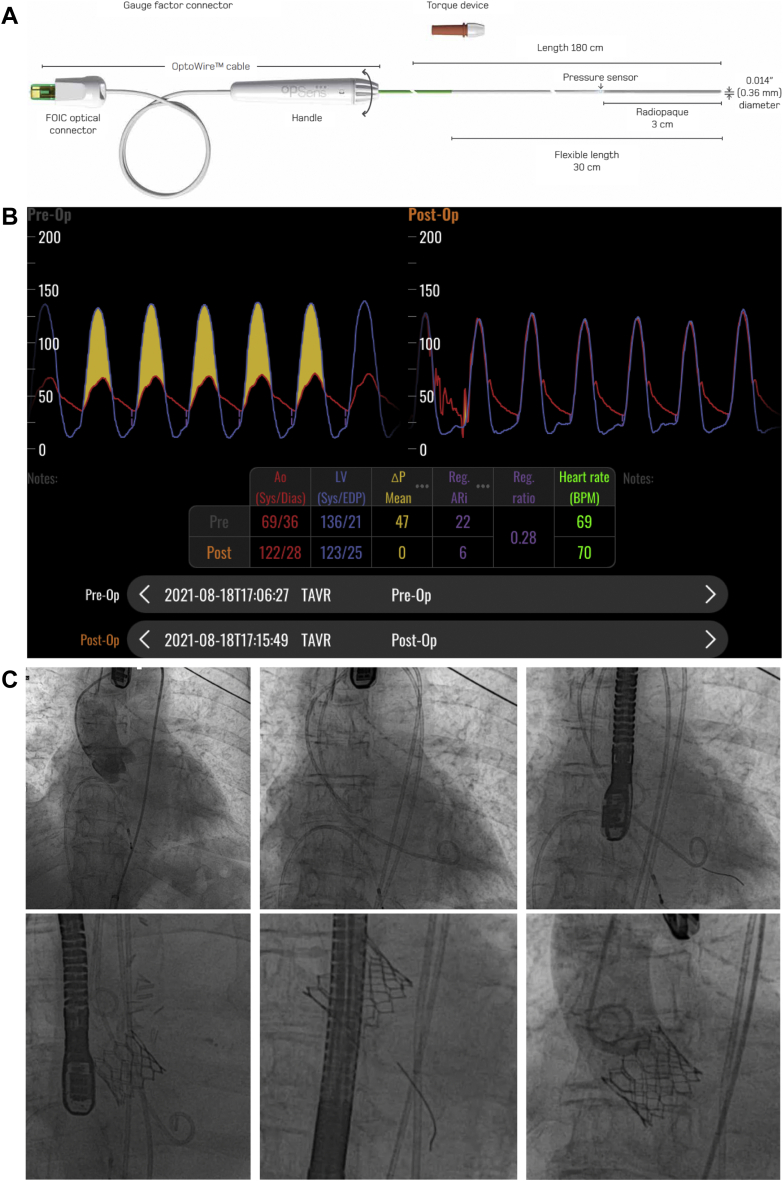
OpSens OptoWire III, monitor display, and procedure steps. (**A**) OpSens OptoWire III 0.014″; (**B**) monitor displaying pre- and post-transcatheter aortic valve replacement (TAVR) measurements; (**C**) summary of the procedure. Before TAVR (upper row), a root angiogram, 2-pigtail gradient, and OpSens OptoWire III-derived gradient were performed. After TAVR (lower row), 2-pigtail gradient, OpSens OptoWire III-derived gradient, and a root angiogram were performed. A transthoracic echocardiogram and a transesophageal echocardiogram were performed both before and after TAVR. FOIC, fiber optic interface connector.

The study devices in the current study are the OpSens OptoWire III (already FDA approved for coronary use) and a novel proprietary monitor and software algorithm that process the acquired data. The OpSens OptoWire III is a 0.014″ pressure guidewire developed to perform endovascular pressure measurements. The OpSens OptoWire III is used in conjunction with the OptoMonitor. The OpSens OptoWire III has 4 main parts: a distal 30-mm tip connected to the sensor housing followed by the intermediate section and the shaft (Figure 1A). The sensor housing is the area where the optical pressure sensor is located and comprises an opening (pressure window) to allow blood contact with the diaphragm of the pressure sensor, just distal to the sensor position. The proximal end of the OpSens OptoWire III is connected to a fiber optic interface cable (OptoWire Cable) that allows connection to the OptoMonitor outside the sterile field. The main function of the OptoMonitor is to measure the pressure via the OpSens OptoWire III pressure sensor, but it also displays different hemodynamic measurements such as fractional flow reserve, Pd/Pa, and diastolic pressure ratio.

A new version of the OptoMonitor software specifically designed for TAVR intervention has been developed. This TAVR-dedicated monitor allows for measurement and display of aortic and left ventricular pressure as well as different metrics derived from them including mean gradient, peak-to-peak gradient, maximum instantaneous gradient, and aortic regurgitation assessment metrics such as aortic regurgitation index^9^ and time-integrated regurgitation index.^10^ Gradients and regurgitation indices are displayed in live and playback screens as well as in the comparison screen (Figure 1B), where users may display hemodynamic measurement before and after TAVR.

### Study design

The current study was a prospective, single-arm, single-center study. The main objective was to establish the degree of agreement related to gradient measurement during TAVR between the OpSens OptoWire III compared with hemodynamic values derived by catheterization and standard TTE and TEE. At the time of the index procedure, subjects underwent hemodynamic assessment before and after TAVR—pre-TAVR: TTE, TEE, standard 2-pigtail 6F (1 aortic, 1 ventricular), and OpSens OptoWire III measurements; post-TAVR: TTE, TEE, standard 2-pigtail 6F (1 aortic, 1 ventricular), and OpSens OptoWire III measurements. Measurements performed with TEE, 2-pigtail, and OpSens OptoWire III were obtained almost simultaneously, while TTE before and after TAVR were performed as close as possible in time. The different steps involved within the study are summarized in the Supplemental Appendix. The OpSens OptoWire III was only used for gradient measurements and not for valve delivery or left ventricle pacing. The OpSens OptoWire III was removed for TAVR implantation. The study was approved by the institutional review board, and all patients provided written informed consent. The principal investigator (P.G.) had access to all data. All serious adverse events were site reported. All data were analyzed by the investigators (P.G., B.R.) independent of the sponsors. The TAVR procedure was performed per institution’s standard practice. Following completion of the procedure, subjects were monitored for 24 ​hours. Four independent cardiologists, part of an independent core laboratory (Morristown Medical Center), assessed all TTE and TEE studies. Adverse events and device effects were recorded from enrollment of subjects and throughout the procedure and hospitalization until discharge.

### Study endpoints

The primary endpoint of the current study is the final post-TAVR mean gradient difference and correlation between OpSens OptoWire III and hemodynamic value derived by catheterization. Other important secondary endpoints include pre- and post-TAVR mean gradient difference and correlation between each modality (TTE, TEE, standard catheterization, and OpSens OptoWire III). Major clinical endpoints will be reported according to the Valve Academic Research Consortium 3 document.^11^

### Statistical analysis

Continuous outcome variables are presented as mean ​± ​standard deviation and as median (interquartile range). For continuous variables, we used the Shapiro-Wilk test to examine normality. For categorical outcome variables, relative frequencies were provided. Differences and agreement between standard catheterization and OpSens OptoWire III wire mean gradients before and after TAVR are presented through a Bland-Altman plot as the differences of the 2 measurements plotted against their means.^12^ The 95% limits of agreement between the gradients obtained by catheterization and the OpSens OptoWire III wire were calculated. Secondary endpoints are presented using the same methods. A paired *t* test was used to test for a fixed bias in the mean gradient obtained by the OpSens OptoWire III wire compared with catheterization. Pearson correlation coefficient was calculated to assess correlation among the modalities. Analyses were performed using a commercially available software program (SAS 9.4; SAS Institute). An OpSens software program recorded all the raw measurements performed with the OpSens OptoWire III, and data were store on a password-protected computer.

## Results

### Patients and enrollment

From July 2021 to September 2021, 22 patients consented, and 20 patients were enrolled in the current study. The 2 patients who consented but not enrolled were excluded due to logistic issues within the hybrid operation room, precluding the use of 2 pressure transducers and the appropriate recording of 2 transducers/pigtails pressure measurements. The baseline and procedural characteristics of the studied population are presented in Table 1. The median age was 79 (6.5) years, and 9 (45%) patients were female. The median Society of Thoracic Surgeons score was 2.8% (7.3), and 3 (15%) patients had known atrial fibrillation. Four (25%) patients had an ejection fraction <50%. The mean ejection fraction was 53 ​± ​11%. The Edwards Lifesciences SAPIEN 3/SAPIEN 3 Ultra TAVR system was used in all patients, with 4 (20%) patients receiving a 23-mm valve, 14 (70%) receiving a 26-mm valve, and 2 (10%) receiving a 29-mm valve. All TAVR procedures were performed via an arterial femoral approach. All patients underwent general anesthesia. No balloon predilation or postdilation was performed.

**Table 1:** Baseline and procedural characteristics.

Characteristic	Value
Age, y	79 [6.5]
Female sex	45% (9)
Body mass index, kg/m^2^	27.5 [5.3]
Hypertension	77% (17)
Dyslipidemia	70% (14)
Diabetes	20% (5)
Current smoker	20% (4)
Atrial fibrillation	35% (7)
Previous percutaneous coronary intervention	20% (4)
Previous coronary artery bypass grafting	10% (2)
Previous permanent pacemaker	15% (3)
Previous stroke/transient ischemic attack	10% (2)
Peripheral vascular disease	30% (6)
Severe or O_2_-dependent COPD	5% (1)
Kidney disease on dialysis	10% (2)
STS score, %	2.8 [7.3]
Medication pre-TAVR	
Aspirin	75% (15)
P2Y12 inhibitor	20% (4)
Oral anticoagulation	35% (7)
Angiotensin-converting enzyme inhibitor	25% (5)
Angiotensin II receptor blocker	30% (6)
Beta-blocker	65% (13)
Calcium channel blocker	40% (8)
Nitrate	5% (1)
Diuretic	30% (6)
Sacubitril/valsartan	5% (1)
Statin	75% (15)
Procedural characteristics	
Transfemoral approach	100% (20)
SAPIEN 3 Ultra 23 ​mm	20% (4)
SAPIEN 3 Ultra 26 ​mm	70% (14)
SAPIEN 3 29 ​mm	10% (2)
Balloon predilation	0% (0)
Balloon postdilation	0% (0)
General anesthesia	100% (20)
Rhythm at time of TAVR	
Sinus	85% (17)
Atrial fibrillation	15% (3)

Values are given as % (n) or median [interquartile range].

COPD, chronic obstructive pulmonary disease; STS, Society of Thoracic Surgeons; TAVR, transcatheter aortic valve replacement.

### Gradient measurements before and after TAVR

Table 2 shows hemodynamic assessment before and after TAVR according to different measurement modalities. By TTE, baseline aortic valve area was 0.76 ​± ​0.14 ​cm^2^, mean gradient was 33 ​± ​14 ​mm Hg, and left ventricular ejection fraction was 53 ​± ​11%.

**Table 2:** Hemodynamic measurements before and after transcatheter aortic valve replacement.

	Pre-TAVR measurements	Post-TAVR measurements
Transthoracic echocardiogram		
Aortic valve area, cm^2^	0.76 ​± ​0.14	1.84 ​± ​0.35
Peak velocity, cm/s	362 ​± ​72	165 ​± ​47
Peak gradient, mm Hg	54 ​± ​21	11.7 ​± ​6.9
Mean gradient, mm Hg	33 ​± ​14	5.8 ​± ​3.1
Left ventricle ejection fraction, %	53 ​± ​11	55 ​± ​11
Transesophageal echocardiogram		
Peak velocity, cm/s	376 ​± ​71	166 ​± ​40
Peak gradient, mm Hg	58 ​± ​22	12.2 ​± ​5.6
Mean gradient, mm Hg	34 ​± ​13	5.6 ​± ​2.7
Catheterization with 2 pigtail catheters		
Peak gradient, mm Hg	44 ​± ​22	3.0 ​± ​4.1
Mean gradient, mm Hg	35 ​± ​14	2.2 ​± ​3.5
OpSens OptoWire III		
Peak gradient, mm Hg	40 ​± ​18	1.1 ​± ​2.4
Mean gradient, mm Hg	35 ​± ​14	2.8 ​± ​2.7

Values are mean ​± ​standard deviation.

TAVR, transcatheter aortic valve replacement.

The mean gradient before TAVR derived by using the standard catheterization 2-pigtail technique was similar to the mean gradient using the OpSens OptoWire III (35 ​± ​14 ​mm Hg vs 35 ± 14 ​mm Hg, *P* = 1.00), with a mean absolute difference of 2.2 ​± ​3.5 ​mm Hg and a strong correlation (*r* = 0.96, *P* < .0001; Table 3). After TAVR, the mean gradient derived by the standard catheterization 2-pigtail technique was similar to the mean gradient using the OpSens OptoWire III (2.2 ​± ​3.5 vs 2.8 ​± ​2.7, *P* = .16), with a mean absolute difference of 1.2 ​± ​1.3 ​mm Hg and a strong correlation (*r* = 0.89, *P* < .0001). Patients with the most discrepancy in mean gradient measurement before and after TAVR were in atrial fibrillation at the time of the index procedure and had an ejection fraction <50%.

**Table 3:** Difference and correlation between mean gradient measurements obtained by different modalities before and after transcatheter aortic valve replacement.

Modality	Absolute mean difference^a^	*P* value	Pearson correlation
Pre-TAVR mean gradient			
OpSens vs cath	2.2 ​± ​3.5	1.00	0.96
OpSens vs TEE	3.0 ​± ​3.2	.30	0.96
OpSens vs TTE	7.8 ​± ​7.5	.32	0.70
Cath vs TEE	3.5 ​± ​3.0	.33	0.96
Cath vs TTE	6.7 ​± ​5.1	.20	0.84
TEE vs TTE	5.8 ​± ​6.0	.44	0.81
Post-TAVR mean gradient			
OpSens vs cath	1.2 ​± ​1.3	.16	0.89
OpSens vs TEE	3.1 ​± ​1.9	<.0001	0.61
OpSens vs TTE	3.2 ​± ​1.7	<.0001	0.71
Cath vs TEE	3.9 ​± ​2.0	<.0001	0.55
Cath vs TTE	3.6 ​± ​2.2	<.0001	0.75
TEE vs TTE	1.8 ​± ​1.7	1.00	0.63

Cath, catheterization using 2-pigtail catheters; OpSens, OpSens OptoWire III; TAVR, transcatheter aortic valve replacement; TEE, transesophageal echocardiogram; TTE, transthoracic echocardiogram.

a Values are given as mm Hg ​± ​standard deviation.

**Figure 2:**
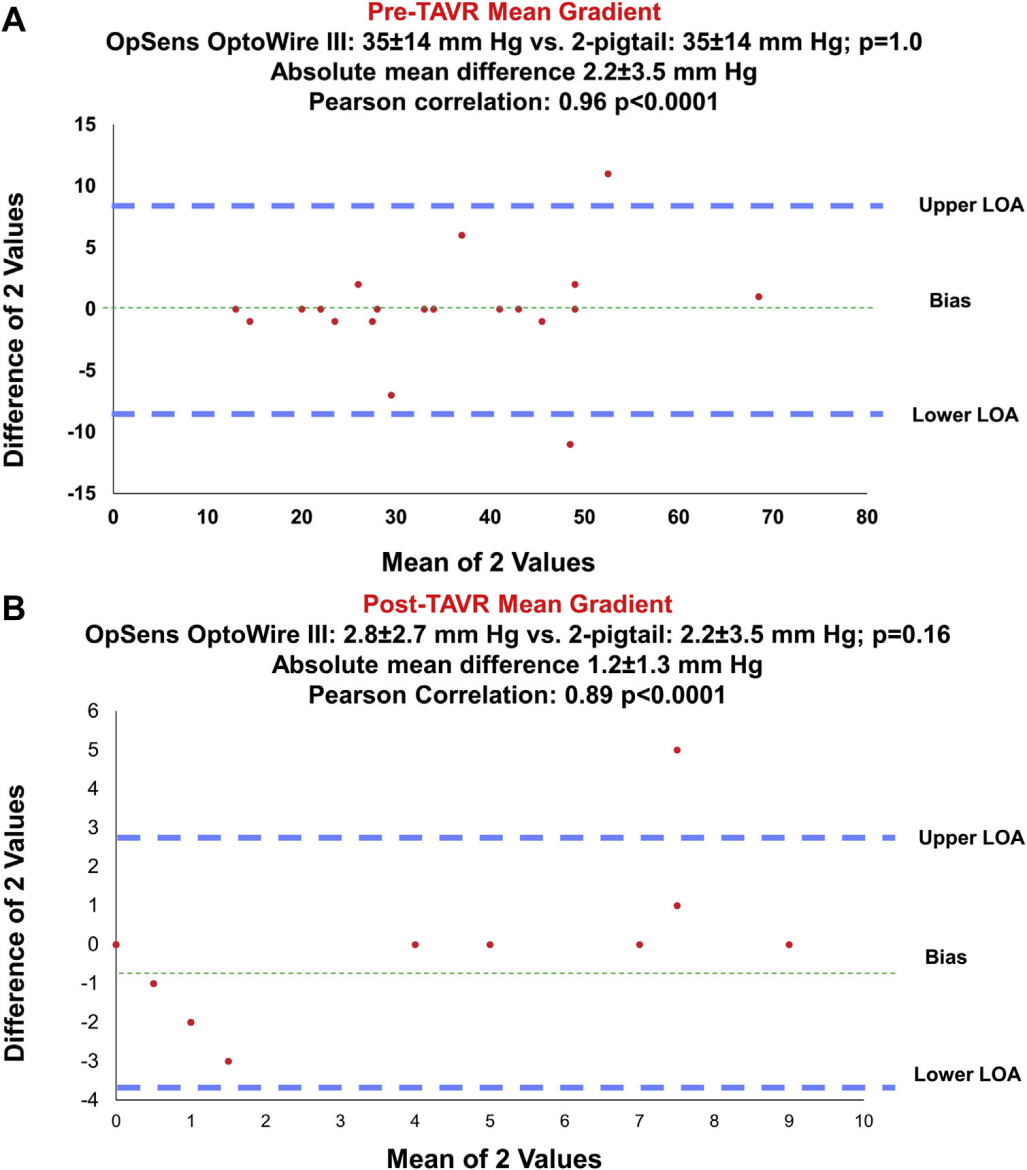
Bland-Altman plot for pre-TAVR and post-TAVR mean gradient measurements obtained with OpSens OptoWire III and by standard catheterization using a 2-pigtail technique. (**A**) Before transcatheter aortic valve replacement (TAVR); (**B**) after TAVR. LOA, limit of agreement.

Difference and correlation between each modality are shown in Table 3. In general, no significant difference and good correlation were observed before TAVR between the different modalities; however, after TAVR, significant differences were noted, especially between echocardiographic measurements (TTE and TEE) and invasive measurement (2-pigtails and OpSens OptoWire III); however, OpSens OptoWire III demonstrated strong correlation and insignificant difference compared with standard 2-pigtail technique both before and after TAVR. Figure 2 shows a Bland-Altman Plot illustrating the difference and correlation between the 2-pigtail technique and the OptoWire III measurement related to the mean gradient before (Figure 2A) and after TAVR (Figure 2B). An expected higher absolute variability at higher pressure was noted, but the mean difference in the pressure gradient derived by the OptoWire and 2-pigtail technique was unrelated to the magnitude of the pressure gradient before TAVR. Conversely, the OptoWire appeared to overestimate lower pressure gradients and underestimate larger pressure gradients compared with the standard 2-pigtail technique after TAVR (*P* = .01).

### Clinical outcomes

At 24 ​hours, no adverse events occurred within the studied population. No adverse events related to the studied devices were reported.

## Discussion

The main findings of the current study are as follows: (1) The OpSens OptoWire III and new TAVR algorithm demonstrated excellent correlation with measurement derived by catheterization using the 2-pigtail catheter; (2) compared with TTE and TEE, the OpSens OptoWire III demonstrated the strongest correlation with catheterization measurement, both before and after TAVR; (3) discrepancies exist between the gradients derived by echocardiogram vs catheterization, with the most pronounced differences observed for gradients derived after TAVR (Central Illustration).

**Central Illustration:**
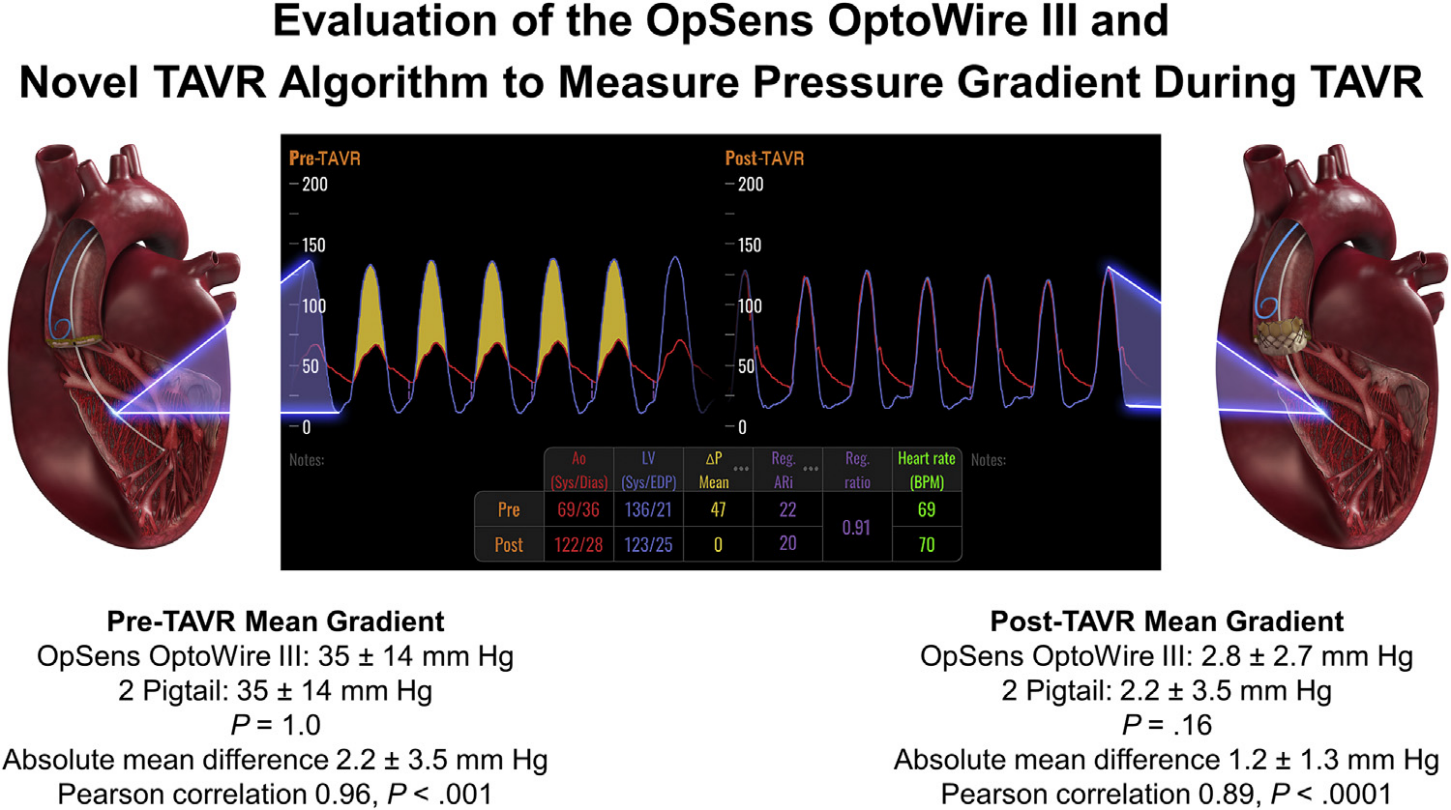
**Evaluation of the OpSens OptoWire III and Novel TAVR Algorithm to Measure Pressure Gradient During TAVR**. The OpSens OptoWire III and new TAVR algorithm demonstrated excellent correlation with measurements derived by a standard double-pigtail technique both before and after TAVR. TAVR, transcatheter aortic valve replacement.

### Correlation with invasive measurements

The OpSens OptoWire III and new TAVR algorithm demonstrated excellent correlation with measurements derived by a standard double-pigtail technique both before and after TAVR. These findings are important and may lead to simplification of the TAVR procedure and expansion of its use for native or prosthetic valve assessment. Indeed, current invasive hemodynamic assessment of aortic valve stenosis or degenerated bioprostheses requires 2 transducer systems and 2 catheters, which could seem cumbersome, and also involves crossing a diseased valve with a relatively large catheter. In the current study, we used the OpSens OptoWire III 0.014″ wire before and after the TAVR procedure. While the use of a 0.014″ wire to cross an aortic valve is not done routinely during a TAVR procedure, a dedicated 0.35″ version has recently been studied in a feasibility study, with promising results (NCT05082337). That being said, the use of a 0.014″ wire, with its dedicated AS algorithm, to assess the severity of AS could be seen as useful for patients with undetermined AS severity or echocardiogram discordance between gradient and valve area. Indeed, the lower profile of the 0.014″ compared with a 0.035″ or a 6F catheter will create less interaction with leaflet coaptation, reducing the risk of device-induced insufficiency or stenosis. Similarly, the lower profile of a 0.014″ wire could be appealing, especially when crossing a diseased valve, with embolism being a concern when using a larger wire or catheter.

Prior work has demonstrated the feasibility, safety, and utility of using a 0.014″ pressure wire to assess hemodynamic severity of AS.^13^, ^14^, ^15^, ^16^ Indeed, Bae et al^13^ reported similar findings using a pressure wire to measure left ventricular pressure and a 5F catheter to measure ascending aortic pressure. They reported similar measurements of gradient between Doppler echocardiography and pressure wire (32.9 ​± ​12.1 ​mmHg), with great correlation (r = 0.741; *P* < .001).^13^ Similarly, 2 other groups described the utility of a 0.014″ pressure wire to help assess AS severity in challenging situations such as low flow state, low ejection fraction, or atrial fibrillation.^15^^,^^16^ Our findings expand on those prior reports and demonstrate the utility of a dedicated algorithm, software, and console to better display the live data to operators and the potential value of its integration within a dedicated 0.035″ stiff TAVR wire (SavvyWire) with intrinsic pacing capability to simplify the TAVR procedure.

### Comparison with echocardiographic measurement

The discrepancy between echocardiogram- and catheter-derived gradients has been previously reported by multiple groups.^17^, ^18^, ^19^ Overestimation of transvalvular gradients from Doppler-derived measurements vs invasive measurements is well described in native AS.^20^^,^^21^ It is mainly attributed to the pressure recovery phenomenon and inherent Bernoulli-equation limitations. The pressure recovery phenomenon occurs because of the downstream increase in the pressure due to the conversion of the kinetic energy to potential energy. These conversions result in overestimation of Doppler gradients compared with catheter gradients. Valve morphology contributes to inflow and outflow gradients and the phenomenon of pressure recovery. It is more pronounced in tunnel-like AS.^22^ It can also be seen in hypertrophic cardiomyopathy, surgically implanted prosthetic valves, and subvalvular or supravalvular stenosis.^23^ Aortic annulus size can also exacerbate the pressure recovery phenomenon.

### Discrepancies between invasive and echocardiographic measurements after TAVR

Recent findings have also suggested that discordance exists between echocardiographic and invasive measurements after TAVR implantation, most likely due to inherent limitations of echocardiographic data acquisition in transvalvular gradient and velocity and other phenomena such as flow recovery after TAVR implantation.^24^, ^25^, ^26^, ^27^ A recent multicenter study of 808 patients demonstrated significant discrepancies between invasive measurement of gradient after TAVR and TTE-derived gradient immediately after TAVR and at discharge.^28^ This discordance was seen in both balloon- and self-expandable valves. Those findings were also confirmed by in vitro testing and modeling, where transvalvular gradient after TAVR implantation was seen to be approximatively 40% higher by TTE Doppler assessment than invasive hemodynamic measurements.^29^ Understanding discrepancy in postintervention measurements from different approaches is important and highlights the need for an accurate, efficient, and reproducible measurement technique to ensure optimal patient outcomes.^30^ This could be especially true when performing a valve-in-valve procedure, where residual elevated gradients are frequent and the need for aggressive dilation after TAVR (bioprosthetic valve fracture) is often required.^31^, ^32^, ^33^, ^34^ Therefore, a measurement technique that can reduce the need for a cumbersome setup (double pigtail, double transducer) and multiple catheter exchanges while improving efficacy and providing high accuracy in gradient measurement would bring incremental value.

### Future directions

The aim of the current study was to validate the capacity of the Fidela optical sensor imbedded within a 0.014″ wire (OpSens OptoWire III) and its novel software/algorithm to assess and display in real time hemodynamics before and after TAVR. While the study successfully demonstrated excellent correlation between the OpSens OptoWire III and the standard double-pigtail technique, integration of this technology within a dedicated 0.035″ TAVR wire used for valve delivery, paired with the capacity to rapid pace the left ventricle and to show continuous hemodynamic measurements, represents the most useful and exciting application of this technology. Such wire, the SavvyWire, is currently under investigation (NCT05082337). Nevertheless, the OpSens OptoWire III 0.014″ wire and its dedicated software could be useful in many cases, such as confirmation of AS severity, invasive dobutamine response assessment, failed bioprosthesis assessment (TAVR or surgical), or even mechanical prosthesis assessment. Finally, integration of aortic regurgitation indices could enhance the value of such technology and potentially marginalize the role of TTE and TEE during TAVR.

### Limitations

The current study represents the first evaluation of the new OpSens TAVR algorithm for pressure assessment before and after TAVR using the 0.014″ OpSens OptoWire III. Nonetheless, several limitations of the present study should be acknowledged. First, our study represents a single-center study experience with a limited number of patients. However, a rigorous methodology and the use of independent core lab assessment for echo-derived gradients made the finding of our study unique. Second, measurements obtained by TEE, double pigtails, and OpSens OptoWire III were performed almost simultaneously; however, a short and variable delay intrinsic to TTE acquisition measurements was present. While the primary goal of the current study was to test the correlation between 2-pigtail technique and OpSens OptoWire III, we do not expect any clinically meaningful difference between TTE and other gradient measurements modalities. Third, only balloon-expandable TAVR valves were used. Results with different TAVR prostheses and among all-comer TAVR patients will be interesting. Fourth, due to the performance of TEE on all patients, general anesthesia was used. While conditions were similar for all measurement modalities, general anesthesia is known to affect valve hemodynamic, and results from TAVR performed under conscious sedation would also be interesting. Importantly, the use of the OpSens OptoWire III does not require general anesthesia and could also be used during conscious sedation. The eventual availability of the dedicated 0.035″ TAVR wire (SavvyWire) offering continuous and live hemodynamic assessment and pacing capability might help to convert sites and operators to adopt a more minimalistic approach with conscious sedation only. Fifth, 3 patients were in atrial fibrillation during the index procedure. Challenges in obtaining gradient is well known when arrhythmia is present. Indeed, in our study, variability in gradient measurements was highest among patients with atrial fibrillation at the time of TAVR and also among patients with a low ejection fraction (<50%). However, assessment through an independent core laboratory insured consistency in measurement technique to minimize variability in data reporting. Finally, the current validation of the OpSens novel TAVR algorithm was performed with a 0.014″ wire and not a 0.035″ wire. That being said, the optical sensor imbedded within the 0.014″ OpSens OptoWire III and the software algorithm integrated within the OpSens console are the same as the one used for the upcoming 0.035″ dedicated TAVR wire (SavvyWire).

## Conclusion

Hemodynamic assessment derived from the 0.014″ OpSens OptoWire III and the new TAVR algorithm demonstrated excellent correlation with measurement derived by 2 pigtails, both before and after TAVR. Integration of this new technology within a dedicated TAVR wire with live hemodynamic assessment could bring meaningful value to TAVR operators.

## Declaration of competing interest

Dr Philippe Genereux is a consulat for Abbott Vascular, Abiomed, BioTrace Medical, Boston Scientific, CARANX Medical, Cardiovascular System Inc, Edwards LifeSciences, GE Healthcare, iRhythm Technologies, Medtronic, OpSens, Pi-Cardia, Puzzle Medical, Saranas, Shockwave, Siemens, Soundbite Medical Inc, Teleflex, and 4C Medical; is an advisor for Abbott Vascular, Abiomed, BioTrace Medical, Edwards LifeSciences, and Medtronic; receives speaker fees from Abbott Vascular, Abiomed, BioTrace Medical, Edwards LifeSciences, Medtronic, and Shockwave; is a primary investigator of the ECLIPSE trial in Cardiovascular System Inc; is a proctor for Edwards LifeSciences; receives research grant from 10.13039/100006520Edwards LifeSciences; is a primary investigator of the PI EARLY-TAVR and PI PROGRESS trials of Edwards LifeSciences; has equity in Soundbite Medical Inc; and is a primary investigator of the PI Feasibility study of 4C Medical. Other authors have no disclosures.

## Supplementary material

Supplemental Appendix Rosters and disclosure information for the technical review team and guideline panel
