# Supplementary material for: Evaluation of the OpSens OptoWire III and Novel TAVR Algorithm to Measure Pressure Gradient During TAVR
Source: J Soc Cardiovasc Angiogr Interv. 2022 May 17;1(4):100309. doi: 10.1016/j.jscai.2022.100309 (PMC11307586; doi:10.1016/j.jscai.2022.100309)
Supplement: Supplemental Appendix — Rosters and disclosure information for the technical review team and guideline panel [file mmc1.docx]

**Supplemental Appendix**

**Hemodynamic Assessment Derived by Catheterization**

Hemodynamic assessment (transvalvular peak-to-peak gradient and mean gradient) was performed using 2 pressure transducers, 2 manifolds, and 2 6F pigtails at baseline pre-transcatheter aortic valve replacement (TAVR) and post-TAVR according to a standardized invasive hemodynamic measurement as below:

**1) Two pigtails measurement pre-TAVR**

- Two table-mounted transducers
- Equalized/zeroed both manifolds
- Two 6F pigtails
- Equalized 2 pigtails in the aorta (kissing pigtails)
- Cross aortic valve with standard wire (straight stiff 0.035”) with AL-1 6F catheter
- Exchange AL-1 6F catheter in the left ventricle (LV) for a 6F pigtail
- Transvalvular gradient recording: 10-second tracings mid-ventricle

**2) OpSens OptoWire III measurement pre-TAVR**

- Advanced OpSens OptoWire III in the 6F LV pigtail
- Equalized OpSens OptoWire III with the 6F LV pigtail
- Transvalvular gradient recording between OpSens OptoWire III and the aortic 6F pigtail 10-second tracings mid-ventricle

Transthoracic echocardiography (TTE) and transesophageal echocardiography (TEE) measurements were done simultaneously for comparison.

**3) TAVR procedure**

- Exchange the OpSens OptoWire III from the LV for standard dedicated pre-curved 0.035” LV wire over 6F pigtail
- Perform TAVR procedure

**4) Two pigtails measurement post-TAVR**

- Over the dedicated 0.035” TAVR wire, bring 6F pigtail in the LV
- Gradient recording between aortic and LV 6F pigtail
- Transvalvular gradient recording: 10-second tracings mid-ventricle

**5) OpSens OptoWire III measurement pre-TAVR**

- Advanced OpSens OptoWire III in the 6F LV pigtail
- Transvalvular gradient recording between OpSens OptoWire III and the aortic 6F pigtail 10-second tracings mid-ventricle

TTE and TEE measurement were done simultaneously for comparison.
